# Association of Improved Periconception Hemoglobin A_1c_ With Pregnancy Outcomes in Women With Diabetes

**DOI:** 10.1001/jamanetworkopen.2020.30207

**Published:** 2020-12-23

**Authors:** Alexander J. F. Davidson, Alison L. Park, Howard Berger, Kazuyoshi Aoyama, Ziv Harel, Eyal Cohen, Jocelynn L. Cook, Joel G. Ray

**Affiliations:** 1 Department of Medicine, University of Toronto, Toronto, Ontario, Canada; 2 ICES, Toronto, Ontario, Canada; 3 Department of Medicine, St Michael’s Hospital, Toronto, Ontario, Canada; 4 Department of Obstetrics and Gynaecology, St Michael’s Hospital, Toronto, Ontario, Canada; 5 Department of Anesthesia and Pain Medicine, The Hospital for Sick Children, Toronto, Ontario, Canada; 6 Child Health Evaluative Sciences, The Hospital for Sick Children, Toronto, Ontario, Canada; 7 Department of Pediatrics, The Hospital for Sick Children, University of Toronto, Toronto, Ontario, Canada; 8 Department of Obstetrics and Gynaecology, University of Ottawa, Ottawa, Ontario, Canada; 9 The Society of Obstetricians and Gynaecologists of Canada, Ottawa, Ontario, Canada

## Abstract

**Importance:**

Prepregnancy diabetes is associated with higher perinatal and maternal morbidity, especially if periconception glycemic control is suboptimal. It is not known whether improved glycemic control from preconception to early pregnancy and midpregnancy periods can reduce the risk of adverse perinatal and maternal outcomes.

**Objective:**

To determine whether a net decline in glycated hemoglobin A_1c_ (HbA_1c_) from preconception to the first half of pregnancy is associated with a lower risk of adverse outcomes for mother and child.

**Design, Setting, and Participants:**

This population-based cohort study was completed in all of Ontario, Canada, from 2007 to 2018. Included were births among women with prepregnancy diabetes whose HbA_1c_ was measured within 90 days preconception and again from conception through 21 weeks completed gestation (early pregnancy to midpregnancy). Statistical analysis was performed from July to September 2020.

**Exposures:**

Net decrease in HbA_1c_ from preconception to early pregnancy and midpregnancy.

**Main Outcomes and Measures:**

The main outcome was a congenital anomaly from birth to age 1 year. Other outcomes included preterm birth or perinatal mortality among offspring as well as severe maternal morbidity (SMM) or death among mothers. Adjusted relative risks (aRRs) were calculated per 0.5% absolute net decline in HbA_1c_ from preconception up to early pregnancy and midpregnancy, adjusting for maternal age at conception, preconception HbA_1c_ and hemoglobin concentration, and gestational age at HbA_1c_ measurement.

**Results:**

A total of 3459 births were included, with a mean (SD) maternal age of 32.6 (5.0) years at conception. Overall, the mean (SD) HbA_1c_ decreased from 7.2% (1.6%) preconception to 6.4% (1.1%) in early pregnancy to midpregnancy. There were 497 pregnancies (14.4%) with a congenital anomaly, with an aRR of 0.94 (95% CI, 0.89-0.98) per 0.5% net decrease in HbA_1c_, including for cardiac anomalies (237 infants; aRR, 0.89; 95% CI, 0.84-0.95). The risk was also reduced for preterm birth (847 events; aRR, 0.89; 95% CI, 0.86-0.91). SMM or death occurred among 191 women (5.5%), with an aRR of 0.90 (95% CI, 0.84-0.96) per 0.5% net decrease in HbA_1c_.

**Conclusions and Relevance:**

These findings suggest that women with prepregnancy diabetes who achieve a reduction in HbA_1c_ may have improved perinatal and maternal outcomes. Further study is recommended to determine the best combination of factors, such as lifestyle changes and/or glucose-lowering medications, that can influence periconception HbA_1c_ reduction.

## Key Points

**Question:**

Among women with prepregnancy diabetes, is improved glycemic control, from preconception to early pregnancy to midpregnancy, associated with reduced risk of adverse perinatal and maternal outcomes?

**Findings:**

In this population-based cohort study of 3459 births among women with prepregnancy diabetes, a reduced risk was found for congenital anomalies, preterm birth, perinatal mortality, and severe maternal morbidity per 0.5% net absolute decline in serum glycated hemoglobin A_1c_ from preconception up to early pregnancy to midpregnancy.

**Meaning:**

These findings suggest that women with prepregnancy diabetes who achieve a reduction in glycated hemoglobin A_1c_ from preconception up to early pregnancy to midpregnancy may have improved perinatal and maternal outcomes.

## Teaser

This cohort study examines whether there is an association between a reduction in hemoglobin A_1c_ during the first half of pregnancy and both perinatal and maternal outcomes among women with prepregnacy diabetes in Ontario, Canada.

## Introduction

Among women with prepregnancy diabetes , serum glycated hemoglobin A_1c_ (HbA_1c_) provides a measure of mean blood glucose control over the preceding 90 days.^1^ Women with diabetes and an elevated preconception HbA_1c_ are at higher risk of severe maternal morbidity (SMM),^2^ and their infants are more prone to congenital anomalies,^3,4^ especially cardiac malformations,^5^ as well as preterm birth and death.^6^

In women with prepregnancy diabetes, preconception interventions that promote glycemic control are thought to lower the risk of major congenital anomalies and preterm birth.^7,8^ However, it is not known whether improved glycemic control between the preconception and early pregnancy to midpregnancy periods can reduce the risk of adverse perinatal and maternal outcomes. Accordingly, the current study was undertaken among women with prepregnancy diabetes to determine whether a net decrease in HbA_1c_ from preconception to the first half of pregnancy (hereafter, termed early pregnancy to midpregnancy) is associated with a lower risk of adverse outcomes for mother and child.

## Methods

This population-based cohort study used existing data sets linked by unique encoded identifiers and analyzed at ICES, Ontario, Canada. The Canadian Institute for Health Information’s Discharge Abstract Database (CIHI-DAD) was used to identify all hospital live births and stillbirths in Ontario, where health care is universal, from March 2007 to September 2018 (the available time period for the data). Gestational age in the CIHI-DAD is estimated from the best record in the medical record, largely by ultrasound dating.^9^ In Ontario, at least 95% of pregnancies have an ultrasound.^10^ The Ontario Laboratories Information System contains the majority of outpatient testing in the province from March 2007 to December 2017. We also used the Better Outcomes Registry and Network (BORN) database, the Ontario Diabetes Data set, the Registered Persons Database, Statistics Canada census data, the Ontario Health Insurance Plan (OHIP) Claims Database, and the Immigration, Refugees and Citizenship Canada Permanent Resident Database. Specifics about databases and diagnosis codes are included in eTable 1 in the Supplement. The use of data in this project was authorized under section 45 of Ontario’s Personal Health Information Protection Act, which does not require review by a research ethics board and waives informed patient consent. This study follows the Strengthening the Reporting of Observational Studies in Epidemiology (STROBE) reporting guideline.

### Participants

Eligible participants were all women with prepregnancy diabetes who had a live birth or stillbirth in an Ontario hospital from 21weeks’ gestation onward and whose HbA_1c_ was measured within 90 days preconception (preconception period), and again between conception and 21 weeks’ gestation (early pregnancy to midpregnancy period). Prepregnancy diabetes was based on either inclusion in the Ontario Diabetes Data set before the index pregnancy, and/or if a woman’s preconception HbA_1c_ was greater than 6.4% (to convert to proportion of total hemoglobin, multiply by 0.01).^11^ Excluded were those younger than 16 years or older than 50 years at conception, non–Ontario residents, those without a valid OHIP number or who were otherwise ineligible for OHIP, and women who gave birth or died prior to 21 weeks’ gestation.

### Exposures

Preconception HbA_1c_ was measured any time in the 90-day period before the estimated date of conception, reflective of the standard lifespan of a red blood cell^12^ and mean blood glucose concentration.^11^ Early pregnancy to midpregnancy HbA_1c_ was measured from the estimated date of conception through 21 weeks’ completed gestation. The upper limit of 21 weeks was chosen for several reasons. It is a typical starting point for defining a stillbirth, and most in utero sonographic screening for structural anomalies is completed by this gestational age.^12^ HbA_1c_ tends to change by less than 0.2% between conception and 21 weeks ,^2^ preceding the potential physiological decline in HbA_1c_ that occurs by early pregnancy to midpregnancy.^13,14^ If a woman had more than 1 HbA_1c_ test in the preconception period, the earliest test was used; and if she had more than 1 HbA_1c_ test in the early pregnancy to midpregnancy period, then the latest was used.

As recommended by national diabetes groups,^11,15^ we evaluated HbA_1c_ as an absolute percentage of total hemoglobin using standards set by the International Federation of Clinical Chemistry.^1^ Under Ontario public health regulations, HbA_1c_ assays are monitored for their precision and must be certified annually by the US National Glycohemoglobin Standardization Program.^16^

### Outcomes

#### Perinatal Outcomes

The main perinatal outcome was any congenital anomaly, diagnosed in a live-born infant from birth up to 365 days thereafter, and in a stillborn fetus at the time of the stillbirth. Anomalies detected as an inpatient were based in the *International Classification of Diseases, Tenth Revision, Canada* (*ICD-10-CA*) codes, and those detected as an outpatient were based on an *International Classification of Diseases, Ninth Revision* (*ICD-9*) code billed by a consultant pediatrician. Omitted were congenital anomalies with a concomitant chromosomal disorder, which are unrelated to glycemic control. As they are a common type of congenital anomaly and are associated with prepregnancy diabetes,^8^ an additional outcome comprised any cardiac anomaly, in the absence of a chromosomal disorder, detected in the first year of life (eTable 1 in the Supplement). The outcome of cardiac anomalies was rerun excluding patent ductus arteriosus (*ICD-10-CA* Q25.0), which is associated with prematurity.

Other perinatal outcomes included preterm birth at less than 37 weeks’ gestation, clinician-initiated (iatrogenic) preterm birth at less than 37 weeks’ gestation, spontaneous preterm birth at less than 37 weeks’ gestation, and extreme preterm birth at less than 32 weeks’ gestation, each among live-born infants, as well as perinatal death, namely a stillbirth or a neonatal death at less than 28 days of life.

#### Maternal Outcomes

The main maternal study outcome was SMM or death arising from after 21 weeks’ gestation until the end of the conventional 42-day postpartum period. An additional maternal outcome was SMM or death arising from the index birth up to 42 days post partum. SMM is a composite outcome made up of approximately 40 indicators arising in pregnancy, during labor, or post partum (see eTable 1 in the Supplement). SMM is a validated proxy for both maternal near miss and maternal mortality, as well as prolonged hospital length of stay, and it can be efficiently ascertained using population-based health care data.^15^

### Statistical Analysis

First, the continuous association between preconception HbA_1c_ and the expected higher probability of a congenital anomaly was plotted using modified Poisson regression with a robust error variance. This approach accounts for the possibility of more than 1 birth per woman.^17^ The calculated probability was adjusted for maternal age at conception and the hemoglobin concentration closest to the preconception HbA_1c_ measurement, the latter reflective of maternal anemia, which may prolong red cell lifespan.^18^

Next, the association between preconception HbA_1c_ and each study outcome was assessed by modified Poisson regression, and a relative risk (RR) was calculated per 0.5% absolute increase in HbA_1c_. A 0.5% absolute increment was chosen because it reflects a clinically important change in HbA_1c_.^19^ RRs were adjusted for maternal age and hemoglobin concentration. To enable consistent model convergence, absolute risk differences (ARDs) were calculated using an adapted approach to logistic regression analysis developed by Austin,^20^ based on marginal probabilities of the outcome of interest, also referred to as population-average (mean) probabilities of success for participants with and without exposure. The 95% CIs were estimated therein by bootstrapping with resampling 1000 times.^20^ Otherwise, RRs from the modified Poisson regression were identical to those from the adapted logistic regression method by Austin.

Third, the continuous association between the net decrease in HbA_1c_ from preconception to early pregnancy to midpregnancy and the estimated probability of any congenital anomaly was plotted using modified Poisson regression, adjusted for maternal age at conception, preconception HbA_1c_, gestational age at HbA_1c_ measurement in early pregnancy to midpregnancy, and the hemoglobin concentration closest to the preconception HbA_1c_ measurement. In the corresponding main model, for each perinatal and maternal outcome, RR and ARD and were calculated per 0.5% absolute net decrease in HbA_1c_ between the preconception and early pregnancy to midpregnancy periods, and adjusted for the same aforementioned covariates.

Maternal obesity is an important factor associated with many adverse perinatal and maternal outcomes commonly seen in women with prepregnancy diabetes.^3,21^ Accordingly, among a limited subset of women whose prepregnancy body mass index (BMI, calculated as weight in kilograms divided by height in meters squared) was available in the BORN database, BMI was adjusted for in the model of preconception HbA_1c_ and adverse perinatal and maternal outcomes (additional analysis 1) as well as in the main model of a 0.5% net change in HbA_1c_ associated with each perinatal and maternal outcome (additional analysis 2). An HbA_1c_ less than 6.5% has been proposed as the ideal target value for reducing adverse pregnancy outcomes.^22^ Accordingly, the main model was also stratified by those whose preconception HbA_1c_ was less than 6.4% or greater than or equal to 6.4% (additional analysis 3). Finally, because glycemic control in the period of organogenesis should have the greatest association with congenital anomaly risk,^4,5^ the main model was repeated, with RRs calculated per 0.5% absolute net change in HbA_1c_ between the preconception period and the period limited to 3 to 12 weeks’ gestation (additional analysis 4).

Statistical significance was set at 2-sided *P* < .05, and analyses were planned a priori. Statistical analyses were performed using SAS statistical software version 9.4 for UNIX (SAS Institute) from July to September 2020.

## Results

A total of 3459 pregnancies were included among women with prepregnancy diabetes. HbA_1c_ was measured at a mean (SD) of 44.4 (25.5) days before conception, and then at 13.5 (5.4) weeks’ gestation in early pregnancy to midpregnancy. The mean (SD) maternal age was 32.6 (5.0) years, 1310 women were nulliparous (37.9%), and 65 pregnancies (1.9%) resulted in a stillbirth or live birth with death at less than 28 days (Table 1). More than one-third of births were to immigrant women. The rate of chronic hypertension was 7.9%, and 16.5% of women had a preconception hemoglobin concentration less than 12 g/dL (to convert hemoglobin to grams per liter, multiply by 10.0).

**Table 1.** Characteristics of 3459 Births From Women With Prepregnancy Diabetes Who Underwent Hemoglobin A_1c_ Testing Both Preconception and in Early Pregnancy to Midpregnancy

Characteristic	Participants, No. (%)
Maternal, at the time of conception	
Age, y	
Mean (SD)	32.6 (5.0)
16-19	21 (0.6)
20-29	907 (26.2)
30-39	2243 (64.8)
40-50	288 (8.3)
Parity	
Median (IQR)	1 (0-1)
Nulliparous	1310 (37.9)
Parous	1293 (37.4)
Unknown	856 (24.7)
Prepregnancy BMI, mean (SD)^a^	30.3 (7.4)
Maternal world region of origin	
Canada or long-term resident of Canada	2240 (64.8)
South Asia	523 (15.1)
East Asia/Pacific	214 (6.2)
Caribbean	104 (3.0)
Sub-Saharan Africa	102 (2.9)
Middle East/North Africa	97 (2.8)
Hispanic America	90 (2.6)
Western Nations/Europe	89 (2.6)
Residing in the lowest income quintile area	932 (26.9)
Rural or unknown residence	276 (8.0)
Maternal, within 1 y prior to conception	
Substance or tobacco use disorder	50 (1.4)
Chronic hypertension	272 (7.9)
Kidney disease	46 (1.3)
Serum creatinine, mean (SD), mg/dL^b^	0.664 (0.137)
At the index birth	
Gestational age, mean (SD), wk	37.0 (2.3)
Multiple gestation	79 (2.3)
Stillbirth or live-born death at <28 d	65 (1.9)
Preterm birth <37 wk gestation	847 (24.5)
Extreme preterm birth <32 wk gestation	108 (3.1)
Preconception, in the 90-d period before the estimated date of conception	
Hemoglobin concentration, mean (SD), g/dL	12.97 (1.13)
Hemoglobin A_1c_, %	
Mean (SD)	7.2 (1.6)
<5.8	429 (12.4)
5.8-6.4	781 (22.6)
>6.4	2249 (65.0)
Days before conception at which hemoglobin A_1c_ was measured, mean (SD), No.	44.4 (25.5)
Early pregnancy to midpregnancy, from the estimated date of conception up to 21 wk gestation	
Hemoglobin concentration, mean (SD), g/dL	12.38 (1.09)
Hemoglobin A_1c_, %	
Mean (SD), %	6.4 (1.1)
<5.8	974 (28.2)
5.8-6.4	1187 (34.3)
>6.4	1298 (37.5)
Weeks at which hemoglobin A_1c_ was measured in pregnancy, mean (SD), No.	13.5 (5.4)

Abbreviations: BMI, body mass index (calculated as weight in kilograms divided by height in meters squared); IQR, interquartile range.

SI conversion factors: To convert serum creatinine to μmol/L, multiply by 88.4; hemoglobin concentration to g/L, multiply by 10.0; and hemoglobin A_1c_ to proportion of total hemoglobin, multiply by 0.01.

^a^ Among 587 pregnancies with a recorded prepregnancy BMI.

^b^ Among 3079 pregnancies with a measured prepregnancy serum creatinine.

### Preconception HbA_1c_ and Adverse Perinatal Outcomes

**Figure 1.**
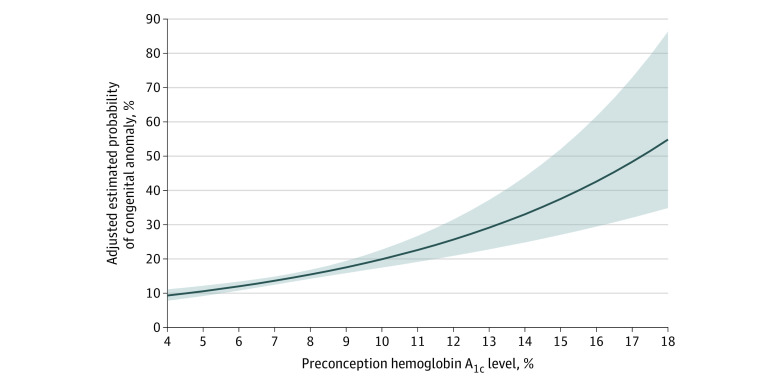
Adjusted Risk of an Infant Congenital Anomaly Diagnosed in the First Year of Life in Association With Maternal Preconception Hemoglobin A_1c_ Among Women With Prepregnancy Diabetes Data are presented as an absolute risk (solid line) and 95% CIs (shaded areas). Risks are adjusted for maternal age at conception and hemoglobin concentration closest to the time of preconception HbA_1c_ measurement. Among 497 infants with a documented congenital anomaly in the first year of life, 313 (63%) were diagnosed in the index birth hospitalization and 185 (37%) diagnosed thereafter. One multifetal pregnancy had 1 infant diagnosed in the birth hospitalization and the other thereafter.

There were 497 pregnancies (14.4%) affected by a congenital anomaly (shown grouped by their *ICD-10-CA* codes in eTable 2 in the Supplement). There was a curvilinear increase in the adjusted probability of a congenital anomaly with increasing preconception HbA_1c_ (Figure 1), which was nearly identical to that in the unadjusted model (eFigure 1 in the Supplement). The unadjusted relative risk (RR) of a congenital anomaly was 1.07 (95% CI, 1.04-1.09) per 0.5% absolute increase in preconception HbA_1c_, which was unchanged after adjusting for other covariates, corresponding to an ARD of 0.97% (95% CI, 0.63%-1.30%) (Table 2). There were 237 infants (6.9%) with a congenital cardiac anomaly, with a corresponding adjusted relative risk (aRR) of 1.09 (95% CI, 1.06-1.13) per 0.5% increase in preconception HbA_1c_. Excluding patent ductus arteriosus, the risk of a cardiac anomaly remained unchanged (191 infants [5.5%]; aRR, 1.10; 95% CI, 1.07-1.14). For each 0.5% higher preconception HbA_1c_, there was a higher aRR for preterm birth less than 37 weeks (1.08; 95% CI, 1.06-1.09), extreme preterm birth less than 32 weeks (1.09; 95% CI, 1.04-1.14), and perinatal mortality (1.16; 95% CI, 1.11-1.22) (Table 2). Among 587 women with known prepregnancy BMI, also adjusting for BMI attenuated the association between preconception HbA_1c_ for some perinatal outcomes (additional analysis 1, eTable 3 in the Supplement).

**Table 2.** Risk of Adverse Perinatal and Maternal Outcomes vs 0.5% Absolute Higher Preconception Maternal Hemoglobin A_1c_ Concentration Among Women With Prepregnancy Diabetes

Outcome	Participants, No. (%)	Relative risk (95% CI)		Adjusted absolute risk difference, % (95% CI)^a^
		Unadjusted	Adjusted^a^	
**Perinatal**				
Congenital anomaly^b^				
Any	497 (14.4)	1.07 (1.05-1.09)	1.07 (1.04-1.09)	0.97 (0.63-1.30)
Cardiac	237 (6.9)	1.10 (1.07-1.13)	1.09 (1.06-1.13)	0.64 (0.40-0.86)
PTB				
Any PTB <37 wk	847 (24.5)	1.08 (1.07-1.10)	1.08 (1.06-1.09)	2.06 (1.61-2.53)
Clinician-initiated live-born PTB <37 wk^c^	629 (18.2)	1.08 (1.07-1.10)	1.08 (1.07-1.10)	1.63 (1.26-2.03)
Spontaneous live-born PTB <37 wk^c^	179 (5.2)	1.05 (1.01-1.09)	1.05 (1.01-1.09)	0.21 (0.00-0.42)
Extreme PTB <32 wk	108 (3.1)	1.08 (1.03-1.13)	1.09 (1.04-1.14)	0.27 (0.10-0.42)
Stillbirth or neonatal death <28 d post partum^d^	65 (1.9)	1.15 (1.10-1.21)	1.16 (1.11-1.22)	0.28 (0.18-0.37)
**Maternal**				
Severe maternal morbidity or death arising from 21 wk gestation ≤42 d post partum	191 (5.5)	1.12 (1.08-1.15)	1.12 (1.09-1.15)	0.63 (0.45-0.82)
Severe maternal morbidity or death arising from the index birth to ≤42 d post partum	91 (2.6)	1.07 (1.02-1.13)	1.08 (1.03-1.14)	0.20 (0.05-0.33)

Abbreviation: PTB, preterm birth.

^a^ Adjusted for maternal age at conception and hemoglobin concentration closest to the time of preconception hemoglobin A_1c_ measurement.

^b^ Excluding any chromosomal anomaly, among 3452 pregnancies.

^c^ Among 3411 pregnancies after excluding deliveries resulting in a stillbirth.

^d^ Among 3456 pregnancies after excluding live births without postpartum data.

### Preconception HbA_1c_ and Adverse Maternal Outcomes

There were 191 pregnancies (5.5%) affected by SMM or death from 21 weeks’ gestation up to 42 days post partum with an aRR of 0.90 (95% CI, 0.84-0.96) per 0.5% net decrease in HbA_1c_, and 43 (1.2%) pregnancies had more than one SMM indicator. The aRR was 1.12 (95% CI, 1.09-1.15) per 0.5% increase in preconception HbA_1c_, corresponding to an ARD of 0.63% (95% CI, 0.45%-0.82%) (Table 2). A similar finding was seen for SMM or death from birth up to 42 days post partum (Table 2). Further adjusting for prepregnancy BMI, the aRR for SMM or death from 21 weeks’ gestation up to 42 days post partum remained similar (additional analysis 1, eTable 3 in the Supplement).

### Net Change in HbA_1c_ and Adverse Perinatal Outcomes

**Figure 2.**
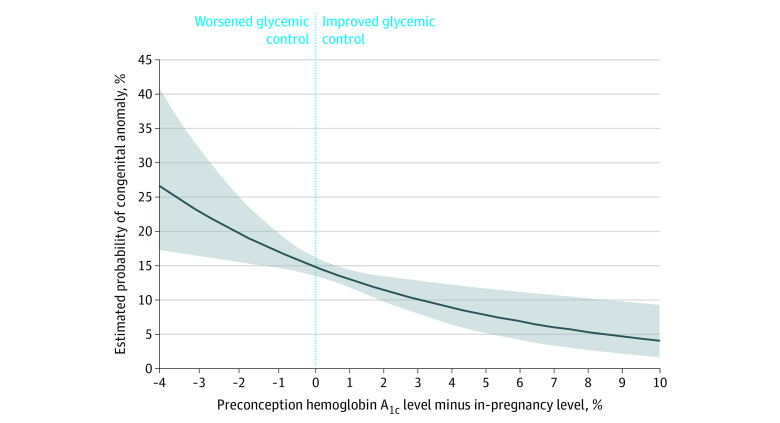
Risk of an Infant Congenital Anomaly Diagnosed in the First Year of Life vs the Net Difference in the Maternal Hemoglobin A_1c_ (HbA_1c_) Between the Preconception and Early Pregnancy to Midpregnancy Periods Among Women With Prepregnancy Diabetes (Main Model) Data are presented as an absolute risk (solid line) and 95% CIs (shaded region). Risks are adjusted for preconception HbA_1c_, maternal age at conception, hemoglobin concentration closest to the time of preconception HbA_1c_ measurement, and the gestational age of HbA_1c_ measurement in the early pregnancy to midpregnancy period.

The mean (SD) HbA_1c_ concentration was 7.2% (1.6%) preconception and was 6.4% (1.1%) in early pregnancy to midpregnancy (Table 1). As the net difference in maternal HbA_1c_ improved from preconception to early pregnancy to midpregnancy, a lower adjusted probability of a congenital anomaly was observed (Figure 2). For example, women with a 2.0% absolute net change in HbA_1c_ from preconception to early pregnancy to midpregnancy had an absolute risk of an infant congenital anomaly of 12.0% (95% CI, 14.0%-17.4%), in contrast to a 15.6% absolute risk (95% CI, 10.4%-13.8%) with a 0 net decrease in HbA_1c_ (Figure 2). Each 0.5% absolute net decrease in HbA_1c_ was associated with a 6% relative decrease (aRR, 0.94; 95% CI, 0.89 to 0.98) and a 1% absolute decrease (ARD −0.99%; 95% CI, −1.79% to −0.27%) in any anomaly, as well as an 11% relative decrease in any cardiac anomalies (aRR, 0.89; 95% CI, 0.84 to 0.95) (Table 3). There was a 12% relative decrease in cardiac anomalies excluding patent ductus arteriosus (aRR, 0.88; 95% CI, 0.82 to 0.94). A lower risk of adverse outcomes was observed for all other perinatal outcomes, although not significantly so for extreme preterm birth (aRR, 0.92; 95% CI, 0.82 to 1.02) (Table 3). In a subset of 587 women, further adjusting for BMI yielded a protective association for preterm birth at less than 37 weeks (aRR, 0.87; 95% CI (0.80 to 0.94) and perinatal mortality (aRR, 0.78; 95% CI, 0.62 to 0.99), but not the other outcomes (additional analysis 2, eTable 4 in the Supplement).

**Table 3.** Risk of Adverse Perinatal and Maternal Outcomes per 0.5% Absolute Net Decline in Maternal Hemoglobin A_1c_ Between the Preconception and Early Pregnancy to Midpregnancy Periods Among Women With Prepregnancy Diabetes (Main Model)

Outcome	Participants, No. (%)	Relative risk (95% CI)		Adjusted absolute risk difference, % (95% CI)^b^
		Adjusted^a^	Further adjusted^b^	
**Perinatal**				
Congenital anomaly^c^				
Any	497 (14.4)	0.95 (0.91 to 0.99)	0.93 (0.89 to 0.98)	−0.99 (−1.79 to −0.27)
Cardiac	237 (6.9)	0.90 (0.85 to 0.95)	0.89 (0.84 to 0.95)	−0.89 (−1.51 to −0.35)
PTB				
Any PTB <37 wk	847 (24.5)	0.92 (0.89 to 0.94)	0.89 (0.86 to 0.91)	−3.20 (−4.26 to −2.30)
Clinician-initiated live-born PTB <37 wk^d^	629 (18.2)	0.92 (0.89 to 0.95)	0.89 (0.86 to 0.93)	−2.28 (−3.17 to −1.47)
Spontaneous live-born PTB <37 wk^d^	179 (5.2)	0.91 (0.85 to 0.98)	0.87 (0.80 to 0.95)	−0.83 (−1.52 to −0.30)
Extreme PTB <32 wk	108 (3.1)	0.91 (0.83 to 1.00)	0.92 (0.82 to 1.02)	−0.30 (−0.79 to 0.07)
Stillbirth or neonatal death <28 d post partum^e^	65 (1.9)	0.85 (0.78 to 0.94)	0.86 (0.77 to 0.97)	−0.36 (−0.82 to −0.06)
**Maternal**				
Severe maternal morbidity or death arising from 21 wk gestation ≤42 d post partum	191 (5.5)	0.94 (0.89 to 1.00)	0.90 (0.84 to 0.96)	−0.64 (−1.20 to −0.18)
Severe maternal morbidity or death arising from the index birth ≤42 d post partum	91 (2.6)	0.95 (0.87 to 1.05)	0.89 (0.79 to 1.00)	−0.35 (−0.86 to 0.00)

Abbreviation: PTB, preterm birth.

^a^ Adjusted for preconception hemoglobin A_1c_.

^b^ Adjusted for preconception hemoglobin A_1c_, maternal age at conception, hemoglobin concentration closest to the time of preconception hemoglobin A_1c_ measurement and the gestational age of hemoglobin A_1c_ measurement in the early pregnancy to midpregnancy period.

^c^ Excluding any chromosomal anomaly, among 3452 pregnancies.

^d^ Among 3411 pregnancies after excluding deliveries resulting in a stillbirth.

^e^ Among 3456 pregnancies after excluding live births without postpartum data.

After stratifying the main model by preconception HbA_1c_, the absolute risk of each perinatal outcome was generally higher among women whose preconception HbA_1c_ was greater than or equal to 6.4% than those whose preconception A_1c_ was less than 6.4% (additional analysis 3, eFigure 2 in the Supplement). For example, the respective rates of preterm birth were 28.3% and 16.3%, and perinatal death 2.4% and 0.8%. The corresponding protective effect per 0.5% net HbA_1c_ reduction was more pronounced in those whose preconception HbA_1C_ was greater than or equal to 6.4% (eFigure 2 in the Supplement).

Among the 1424 births to women whose in-pregnancy HbA_1c_ was restricted to between 3 and 12 weeks’ gestation, the 14.1% rate of congenital anomalies was similar to that seen in the entire cohort, as was the corresponding aRR (0.90; 95% CI, 0.83-0.96) (additional analysis 4, eTable 5 in the Supplement). The aRR for cardiac congenital anomalies was also significant (aRR, 0.87; 95% CI, 0.80-0.94). For preterm birth and perinatal death, there were fewer events, and the aRRs were not significant (eTable 5 in the Supplement).

### Net Change in HbA_1c_ and Adverse Maternal Outcomes

The risk of SMM or death was reduced in association with each 0.5% net decrease in HbA_1c_ from preconception to early pregnancy to midpregnancy, whether the outcome occurred from 21 weeks’ gestation up to 42 days postpartum (aRR, 0.90; 95% CI, 0.84 to 0.96; ARD, −0.64%; 95% CI, −1.20% to −0.18%), or from birth up to 42 days thereafter (aRR, 0.89; 95% CI, 0.79 to 1.00; ARD, −0.35%; 95% CI, −0.86% to 0.00%) (Table 3). Again, protective associations of a net decrease in HbA_1c_ were largely seen in women whose preconception HbA_1c_ was greater than or equal to 6.4% (additional analysis 3, eFigure 2 in the Supplement). The risk of SMM or death was also lower in the subgroup of women whose in-pregnancy HbA_1c_ was limited to 3 to 12 weeks’ gestation (eTable 5 in the Supplement, additional analysis 4).

## Discussion

In this population-based cohort study of women with prepregnancy diabetes, a net improvement in periconception HbA_1c_ was associated with a reduced risk of an array of adverse perinatal and maternal outcomes, including congenital anomalies, preterm birth, morbidity, and death. This was especially so in women whose preconception HbA_1c_ was more than 6.4%.

Women with a 2.0% absolute net change in HbA_1c_ from preconception to early pregnancy to midpregnancy had an absolute risk of an infant congenital anomaly of 12.0% (95% CI, 14.0%-17.4%), in contrast to a 15.6% absolute risk (95% CI, 10.4%-13.8%) with a 0 net decrease in HbA_1c_ (Figure 2). These results emphasize the importance of improved glycemic control prior to, and soon after, conception. As the benefit was more evident in women whose preconception HbA_1c_ was greater than or equal to 6.4% (eFigure 2 in the Supplement), there may be a floor effect once HbA_1c_ is too low to derive any additional benefit from HbA_1c_ reduction. The benefits of improved HbA_1c_ may translate not only into a reduced risk of congenital anomalies, but also preterm birth and SMM. The most common conditions contributing to SMM include postpartum hemorrhage, puerperal sepsis, and severe preeclampsia.^15^ Periconception maternal glycemic status appears to influence both fetal organogenesis and placentation.^23^ Hence, better HbA_1c_ control would be expected to reduce some of these conditions, and, in turn, SMM. Among women with insulin resistance who are overweight or obese, a recent Italian randomized clinical trial (RCT) found that a lifestyle intervention beginning at 9 to 12 weeks’ gestation improved neonatal outcomes.^24^ Ongoing HbA_1c_ control in the third trimester of pregnancy is also associated with a lower risk of perinatal mortality.^6^ An additional clinically relevant finding is affirmation of the recommended periconceptional HbA_1c_ of less than 6.5%.^22^

### Strengths and Limitations

Our study had several strengths, including its population-based large sample derived from within a universal health care system, and the capture of important clinical outcomes. Although structural variants of hemoglobin, such as hemoglobin S, were known to interfere with older generations of HbA_1c_ assays, this is unlikely to be the case in the past 15 years.^1^ HbA_1c_ minimally decreases in the first and second trimester of pregnancy,^2^ because of the decreased lifespan and enhanced production of red cells.^13^ However, these effects were largely mitigated herein by controlling for preconception HbA_1c_ and the gestational age of HbA_1c_ measurement.

This study had some limitations. It did not include data on induced abortions or miscarriages before 20 weeks’ gestation. In Canada, the proportion of fetuses affected by a congenital anomaly to be subsequently aborted may have increased since the 1990s, following improved prenatal detection of birth defects.^8,25^ In the US, however, induced abortion does not appear to bear any substantial influence on the assessment of risk factors for congenital anomalies.^26^ The current study did not distinguish the degree of severity of anomalies, including both major and minor malformations, but did include those recognized up to the age of 1 year.

We could not account for deficiencies in vitamin B_12_ or iron, each of which may elevate HbA_1c_ by prolonging red cell survival, nor for liver disease, which can lower HbA_1c_. These effects are largely mediated by maternal anemia,^13,14^ which was controlled for herein. Folate deficiency is now rare in Canada since the introduction of folic acid flour fortification in 1998.^27^

Although we attempted to account for the potential impact of maternal obesity on the study findings, prepregnancy BMI was unknown for most women. Upon restricting to those women with a known BMI, the number of congenital anomaly outcome events decreased from 497 to 80, with no longer any associated benefit from HbA_1c_ reduction (eTable 4 in the Supplement). In contrast, for preterm birth at less than 37 weeks, the number of events decreased from 847 to 141, and the effect sizes still favored an HbA_1c_ reduction (aRR, 0.87; 95% CI, 0.80 to 0.94) (eTable 4 in the Supplement). We also lacked information about diet, insulin, and oral hypoglycemic agents, and we could not distinguish between women with type 1 and type 2 diabetes. The in-pregnancy HbA_1c_ was considered from conception up to 21 weeks’ gestation, such that an HbA_1c_ could have been included even though it was measured after the sensitive period of embryogenesis of 3 to 12 weeks’ gestation. Even so, we adjusted for the gestational age at HbA_1c_ measurement, and additional analysis 4 showed a protective association upon limiting the in-pregnancy HbA_1c_ to 3 to 12 weeks’ gestation.

This study required that a woman had an HbA_1c_ measured both before and after conception. At a preconception HbA_1c_ of 5.5%, for example, the probability of a congenital anomaly was 11.3%—well above the 3% to 5% rate seen in the general population.^25^ The rate of SMM or maternal death was also much higher than expected.^15^ Hence, women included in the current study may comprise a select group of women with diabetes who are especially predisposed to adverse events.

## Conclusions

Our findings suggest that improved periconception HbA_1c_ in women with prepregnancy diabetes is associated with a reduced risk of several adverse outcomes. In Canada and the US, almost half of pregnancies are unplanned.^28,29^ Nonadherence to medications in pregnancy is linked to poor health literacy,^30^ and among pregnant women in the US, suboptimal glycemic control is associated with maternal obesity, multiparity, tobacco use, race, and lower rates of college education.^31^ There are several evidence-based recommendations for improving periconception glycemic control.^22^ HbA_1c_ reduction can be achieved by lifestyle changes^32,33^ and access to glucose lowering medications, both of which are mediated by improved access to health care, pregnancy planning information, and advocacy. For logistical and ethical reasons it is unlikely that an RCT can be completed comparing the influence of tight and less-tight periconception glycemic control on maternal and perinatal outcomes. Further study is merited to determine the best combination of factors that can influence periconception HbA_1c_ reduction.

## Notes

Supplement. **eFigure 1.** Unadjusted Risk of an Infant Congenital Anomaly Diagnosed in the First Year of Life in Relation to Maternal Preconception Hemoglobin A1c Among Women With Pre-Pregnancy Diabetes Mellitus **eFigure 2.** Risk of Adverse Maternal and Perinatal Outcomes Per 0.5% Absolute Net Decline in Maternal Hemoglobin A1c Between The Preconception and Early To Mid-Pregnancy Periods Among Women With Pre-Pregnancy Diabetes Mellitus (Additional Analysis 3) **eTable 1.** Variables Used to Define Cohort Entry and Exclusion Criteria, as Well as Study Exposures, Outcomes, Adjustment, and Stratification **eTable 2.** Classification of the 497 Infant Congenital Anomalies Diagnosed In The First Year Of Life **eTable 3.** Risk of Adverse Perinatal and Maternal Outcomes in Relation to a 0.5% Absolute Higher Preconception Maternal Hemoglobin A1c Concentration, Restricted to 587 Women With Pre-Pregnancy Diabetes Mellitus And A Recorded Pre-Pregnancy Body Mass Index (BMI) (Additional Analysis 1) **eTable 4.** Risk of Adverse Perinatal and Maternal Outcomes Per 0.5% Absolute Net Decline in Maternal Hemoglobin A1c Between the Preconception and Early To Mid-Pregnancy Periods, Restricted To 587 Women With Pre-Pregnancy Diabetes Mellitus and a Recorded Pre-Pregnancy Body Mass Index (BMI) (Additional Analysis 2) **eTable 5.** Risk of Adverse Perinatal and Maternal Outcomes Per 0.5% Absolute Net Decline in Maternal Hemoglobin A1c Between the Preconception Period And 3 To 12 Weeks’ Gestation Among 1424 Births in Women With Pre-Pregnancy Diabetes Mellitus (Additional Analysis 4)
