# Supplementary material for: Association of Improved Periconception Hemoglobin A1c With Pregnancy Outcomes in Women With Diabetes
Source: JAMA Netw Open. 2020 Dec 23;3(12):e2030207. doi: 10.1001/jamanetworkopen.2020.30207 (PMC7758806; doi:10.1001/jamanetworkopen.2020.30207)
Supplement: Supplement. — eFigure 1. Unadjusted Risk of an Infant Congenital Anomaly Diagnosed in the First Year of Life in Relation to Maternal Preconception Hemoglobin A1c Among Women With Pre-Pregnancy Diabetes Mellitus eFigure 2. Risk of Adverse Maternal and Perinatal Outcomes Per 0.5% Absolute Net Decline in Maternal Hemoglobin A1c Between The Preconception and Early To Mid-Pregnancy Periods Among Women With Pre-Pregnancy Diabetes Mellitus (Additional Analysis 3) eTable 1. Variables Used to Define Cohort Entry and Exclusion Criteria, as Well as Study Exposures, Outcomes, Adjustment, and Stratification eTable 2. Classification of the 497 Infant Congenital Anomalies Diagnosed In The First Year Of Life eTable 3. Risk of Adverse Perinatal and Maternal Outcomes in Relation to a 0.5% Absolute Higher Preconception Maternal Hemoglobin A1c Concentration, Restricted to 587 Women With Pre-Pregnancy Diabetes Mellitus And A Recorded Pre-Pregnancy Body Mass Index (BMI) (Additional Analysis 1) eTable 4. Risk of Adverse Perinatal and Maternal Outcomes Per 0.5% Absolute Net Decline in Maternal Hemoglobin A1c Between the Preconception and Early To Mid-Pregnancy Periods, Restricted To 587 Women With Pre-Pregnancy Diabetes Mellitus and a Recorded Pre-Pregnancy Body Mass Index (BMI) (Additional Analysis 2) eTable 5. Risk of Adverse Perinatal and Maternal Outcomes Per 0.5% Absolute Net Decline in Maternal Hemoglobin A1c Between the Preconception Period And 3 To 12 Weeks’ Gestation Among 1424 Births in Women With Pre-Pregnancy Diabetes Mellitus (Additional Analysis 4) [file jamanetwopen-e2030207-s001.pdf]

Supplemental Online Content

Davidson AJF, Park AL, Berger H, et al. Association of improved periconception hemoglobin A_{1c} with pregnancy outcomes in women with diabetes. *JAMA Netw Open*. 2020;3(12):e2030207. doi:10.1001/jamanetworkopen.2020.30207

eFigure 1. Unadjusted Risk of an Infant Congenital Anomaly Diagnosed in the First Year of Life in Relation to Maternal Preconception Hemoglobin A1c Among Women With Pre-Pregnancy Diabetes Mellitus
eFigure 2. Risk of Adverse Maternal and Perinatal Outcomes Per 0.5% Absolute Net Decline in Maternal Hemoglobin A1c Between The Preconception and Early To Mid-Pregnancy Periods Among Women With Pre-Pregnancy Diabetes Mellitus (Additional Analysis 3)
eTable 1. Variables Used to Define Cohort Entry and Exclusion Criteria, as Well as Study Exposures, Outcomes, Adjustment, and Stratification
eTable 2. Classification of the 497 Infant Congenital Anomalies Diagnosed In The First Year Of Life
eTable 3. Risk of Adverse Perinatal and Maternal Outcomes in Relation to a 0.5% Absolute Higher Preconception Maternal Hemoglobin A1c Concentration, Restricted to 587 Women With Pre-Pregnancy Diabetes Mellitus And A Recorded Pre-Pregnancy Body Mass Index (BMI) (Additional Analysis 1)
eTable 4. Risk of Adverse Perinatal and Maternal Outcomes Per 0.5% Absolute Net Decline in Maternal Hemoglobin A1c Between the Preconception and Early To Mid-Pregnancy Periods, Restricted To 587 Women With Pre-Pregnancy Diabetes Mellitus and a Recorded Pre-Pregnancy Body Mass Index (BMI) (Additional Analysis 2)
eTable 5. Risk of Adverse Perinatal and Maternal Outcomes Per 0.5% Absolute Net Decline in Maternal Hemoglobin A1c Between the Preconception Period And 3 To 12 Weeks' Gestation Among 1424 Births in Women With Pre-Pregnancy Diabetes Mellitus (Additional Analysis 4)

This supplemental material has been provided by the authors to give readers additional information about their work.

© 2020 Davidson AJF et al. *JAMA Network Open.*

eFigure 1. Unadjusted Risk of an Infant Congenital Anomaly Diagnosed in the First Year of Life in Relation to Maternal Preconception Hemoglobin A1c Among Women With Pre-pregnancy Diabetes Mellitus. Data are presented as an absolute risk (solid black line with corresponding italicized blue values) and 95% confidence intervals (dashed red lines).[a]

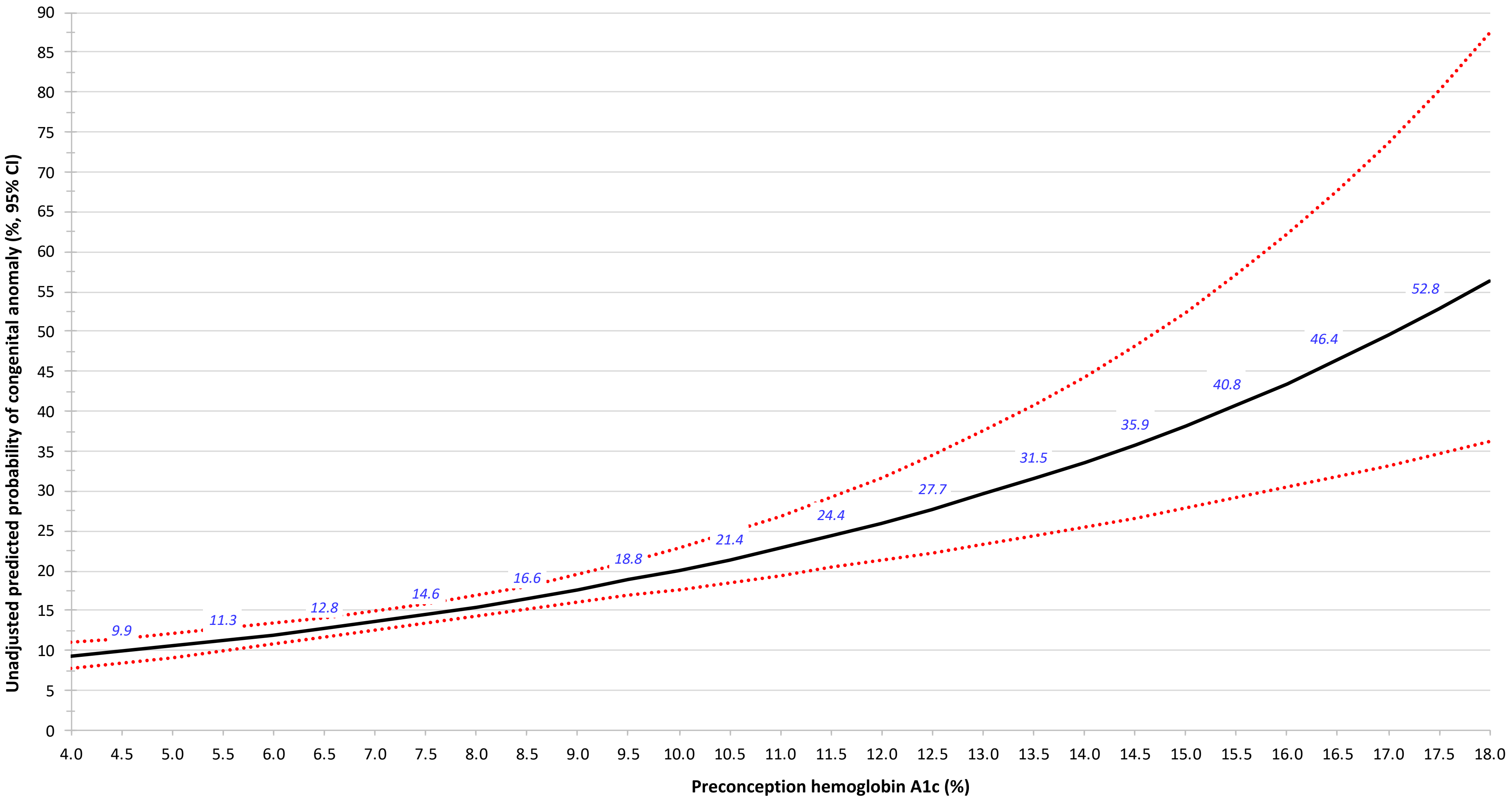

© 2020 Davidson AJF et al. *JAMA Network Open.*

[a] Among 497 infants with a documented congenital anomaly in the first year of life, 313 (63%) were diagnosed in the index birth hospitalization and 185 (37%) diagnosed thereafter. One multifetal pregnancy had one infant diagnosed in the birth hospitalization and the other thereafter

© 2020 Davidson AJF et al. *JAMA Network Open.*

eFigure 2. Risk of Adverse Maternal and Perinatal Outcomes Per 0.5% Absolute Net Decline in Maternal Hemoglobin A1c Between The Preconception and Early To Mid-Pregnancy Periods Among Women With Pre-Pregnancy Diabetes Mellitus (*Additional Analysis 3*). Relative risks are stratified by women whose preconception A1c is < 6.4% (black circles) or ≥ 6.4% (red squares), and also adjusted for preconception A1c, maternal age at conception, hemoglobin concentration closest to the time of preconception A1c measurement, and the gestational age of A1c measurement in the early to mid-pregnancy period.

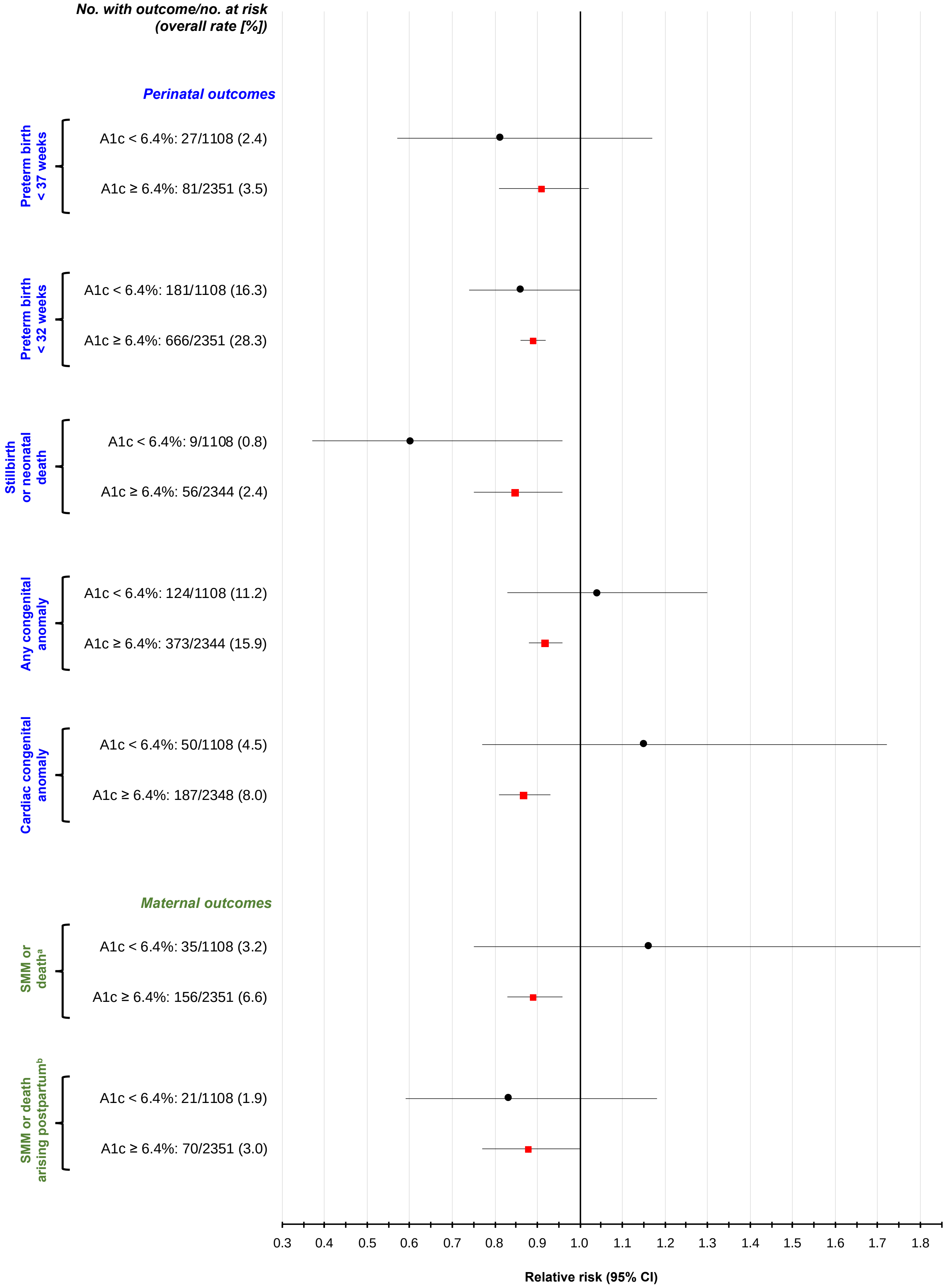

[a] Severe maternal morbidity (SMM) or death arising from 21 weeks' gestation up to 42 days postpartum.

[b] SMM or death arising from the index birth up to 42 days postpartum.

© 2020 Davidson AJF et al. *JAMA Network Open.*

eTable 1. Variables Used to Define Cohort Entry and Exclusion Criteria, as Well as Study Exposures, Outcomes, Adjustment, and Stratification.

**Assessment**	**Timing**	**Disease, procedure or condition**	**ICD-10-CA or CCI codes, or other variables, in CIHI-DAD or MOMBABY**	**OHIP ICD-9 diagnostic codes or fee codes {or other source if in parentheses}**
Inclusion criteria	March 2007 - September 2018	Women with an obstetrically delivered livebirth or stillbirth in Ontario, who had pre-pregnancy diabetes and an A1c measured from minus 90 days up to the estimated date of conception and from conception up to 21 weeks' gestation	Main patient service code indicating "obstetrical delivery" (MOMBABY - includes linked DAD inpatient admission records of delivering mothers and their newborns)	LOINC code "17855-8", "17856-6", "41995-2", "4548-4", "71875-9", "59261-8" in {Ontario Laboratory Information System (OLIS) - includes most outpatient laboratory information in Ontario}<br><br>{Ontario Diabetes Database (ODD) – used to identify women who have been diagnosed with diabetes mellitus}
Exclusion criteria	At the estimated time of conception	Maternal age > 50 years or < 16 years	--	Age {Registered Persons Database (RPDB) contains demographic information and encrypted healthcare numbers for all individuals eligible for OHIP}
	At the time of A1c testing	Non-Ontario resident, invalid OHIP number, otherwise ineligible for OHIP	Invalid OHIP number (DAD delivery record) or a WARN flag in MOMBABY	Province code is not '35', date of birth is missing, or sex is not female {RPDB}
	Prior to 21 weeks' gestation	Stillbirth, livebirth, or death of mother	MOMBABY	Date of death {RPDB}
Study exposure	Up to 90 days before the estimated date of conception	Preconception hemoglobin A1c	--	LOINC code "17855-8", "17856-6", "41995-2", "4548-4", "71875-9", "59261-8" in {OLIS}
	From the estimated date of conception up to 21 completed weeks' gestation	Early to mid-pregnancy hemoglobin A1c	--	LOINC code "17855-8", "17856-6", "41995-2", "4548-4", "71875-9", "59261-8" in {OLIS}
Study outcome	From the index birth up to 1 year following birth	Any congenital anomaly, in the absence of an identified chromosomal abnormality	Q00-Q89, also requiring the exclusion of Q90-Q99	741, 742, 743, 744, 745, 746, 747, 748, 749, 750, 751, 752, 753, 754, 755, 756, 759, also requiring that the diagnosis was billed by a Pediatrician (Specialty Code 26), and also requiring the exclusion of 758

© 2020 Davidson AJF et al. *JAMA Network Open.*

Assessment	Timing	Disease, procedure or condition	ICD-10-CA or CCI codes, or other variables, in CIHI-DAD or MOMBABY	OHIP ICD-9 diagnostic codes or fee codes {or other source if in parentheses}
	Same as above	Any cardiac congenital anomaly, in the absence of an identified chromosomal abnormality	Q20-Q28, also requiring the exclusion of Q90-Q99	745, 746, 747 also requiring that the diagnosis was billed by a Pediatrician (Specialty Code 26), and also requiring the exclusion of 758
	Same as above	Any cardiac congenital anomaly except for patent ductus arteriosus, in the absence of an identified chromosomal abnormality	Q20-Q24, Q251-Q28, also requiring the exclusion of Q90-Q99	745, 746 also requiring that the diagnosis was billed by a Pediatrician (Specialty Code 26), and also requiring the exclusion of 758
	At the index birth	Any preterm birth < 37 weeks' gestation	B_GESTWKS_DEL < 37 in MOMBABY (if missing use M_GESTWKS_DEL)	--
	Same as above	Provider-initiated preterm live birth < 37 weeks' gestation: 1) preterm birth and 2) absence of preterm spontaneous labour and 3) presence of Caesarian delivery or induction of labour	1) Preterm live birth < 37 weeks ' gestation (see above); and 2) absence of O601, O602, O42, O756; and 3) presence of 5MD60* or 5AC30*	--
	Same as above	Spontaneous preterm live birth < 37 weeks' gestation	Preterm live birth < 37 weeks' gestation and not provider-initiated (see above)	--
	Same as above	Very preterm birth < 32 weeks' gestation	Clinical gestation weeks at delivery (DAD newborn record, if present; otherwise use delivery record)	--
	At or within 27 days of the index birth	Stillbirth or neonatal death	O364, Z371, Z373, Z374, Z376, Z377, P95, or discharged not alive within 27 days of birth	Date of death {RPDB}

© 2020 Davidson AJF et al. *JAMA Network Open.*

	From 21 weeks' gestation in the index pregnancy to 42 days following birth	Severe maternal morbidity (SMM) or death	Severe preeclampsia and HELLP syndrome:<br>O14.1, or O14.2<br><br>Eclampsia:<br>O15<br>Cerebral venous thrombosis in pregnancy, or in the puerperium:<br>O22.5, or O87.3<br><br>Acute fatty liver with red blood cell (RBC) transfusion or plasma transfusion:<br>O26.6 + (CIHI BTREDBC = 1 or CIHI BTPLASMA = 1)<br><br>Pulmonary, cardiac, and CNS complications of anaesthesia during pregnancy, the puerperium, or labour and delivery:<br>O29.0, O29.1, O29.2, O89.0, O89.1, O89.2, O74.0, O74.1, O74.2 or O74.3<br><br>Placenta previa with hemorrhage with RBC transfusion:<br>O44.1 + CIHI BTREDBC = 1<br><br>Placental abruption with coagulation defect:<br>O45.0<br><br>Antepartum hemorrhage with coagulation defect:<br>O46.0<br><br>Intrapartum hemorrhage with coagulation defect:<br>O67.0	All cause death in {RPDB}

© 2020 Davidson AJF et al. *JAMA Network Open.*

			Intrapartum hemorrhage with RBC transfusion:<br>O67 + CIHI BTREDBC = 1<br><br>Rupture of the uterus with RBC transfusion, procedures to the uterus or hysterectomy:<br>(O71.0 or O71.1) + any of the following:<br>• CIHI BTREDBC = 1, or<br>• (1.RM.13, 1.KT.51, 5.PC.91.LA or 5.PC.91.HV) + CIHI BTREDBC = 1, or<br>• (5.MD.60.RC, 5.MD.60.RD, 5.MD.60.KE, 5.MD.60.CB or 1.RM.89.LA[a]), or<br>• 1.RM.87.LA-GX<br>[a] **NOTE**: **1.RM.89.LA** is included only if codes 1.PL.74, 1.RS.74 or 1.RS.80 are NOT also present<br><br>Postpartum hemorrhage with RBC transfusion, procedures to the uterus or hysterectomy:<br>O72 + any of the following:<br>• BTREDBC = 1, or<br>• (1.RM.13, 1.KT.51, 5.PC.91.LA or 5.PC.91.HV) + BTREDBC = 1, or<br>• (5.MD.60.RC, 5.MD.60.RD, 5.MD.60.KE, 5.MD.60.CB or 1.RM.89.LA[b]), or<br>• 1.RM.87.LA-GX<br>[b] **NOTE**: **1.RM.89.LA** is included only if codes 1.PL.74, 1.RS.74 or 1.RS.80 are NOT also present	

© 2020 Davidson AJF et al. *JAMA Network Open.*

			Cardiac conditions:<br>O74.2, O89.1, O90.3, I21, I22, I42, I43, I46, I49.0, I50, J81, 1.HZ.09 or 1.HZ.30<br><br>Obstetric shock :<br>O75.1, R57, T80.5 or T88.6<br><br>Septicemia during labour:<br>O75.3<br><br>Complications of obstetric surgery and procedures:<br>O75.4<br><br>Puerperal sepsis:<br>O85<br><br>Obstetric embolism:<br>O88<br><br>Acute renal failure:<br>O90.4, N17, N19 or N99.0<br><br>Death, obstetric, cause unspecified:<br>O95<br><br>Death, obstetric, after 42 days but 1 year after delivery:<br>O96<br><br>Death from sequelae of direct obstetric causes:<br>O97<br>Disseminated intravascular coagulation:<br>D65	

© 2020 Davidson AJF et al. *JAMA Network Open.*

			Sickle cell anemia with crisis:<br>D57.0<br><br>Acute psychosis:<br>F53.1 or F23<br><br>Status epilepticus:<br>G41<br><br>Cerebral edema or coma:<br>G93.6 or R40.2<br><br>Cerebrovascular diseases: subarachnoid and intracranial hemorrhage, cerebral infarction, stroke:<br>I60, I61, I62, I63 or I64<br><br>Status asthmaticus:<br>J45.01, J45.11, J45.81 or J45.91<br><br>Adult respiratory distress syndrome:<br>J80<br><br>Acute abdomen:<br>K35, K37, K65, N73.3 or N73.5<br><br>Hepatic failure:<br>K71 or K72<br><br>Sudden death, death from unspecified cause:<br>R96, R97, R98 or R99<br><br>Assisted ventilation through endotracheal tube:<br>1.GZ.31.CA-ND	

© 2020 Davidson AJF et al. *JAMA Network Open.*

			<u>Assisted ventilation through tracheostomy:</u><br>1.GZ.31.CR-ND<br><br><u>Hysterectomy</u>:<br>5.MD.60.RC, 5.MD.60.RD, 5.MD.60.KE, 5.MD.60.CB, 1.RM.89.LA (exclude if 1.PL.74, 1.RS.74 or 1.RS.80 code also present), 1.RM.87.LA-GX<br><br><u>Dialysis</u>:<br>1.PZ.21<br><br><u>Evacuation of incisional hematoma with RBC transfusion:</u><br>5.PC.73.JS + CIHI BTREDBC = 1<br><br><u>Repair of bladder, urethra, or intestine:</u><br>5.PC.80.JR, 1.NK.80, 1.NM.80<br><br><u>Procedures to the uterus/pelvic vessels with RBC transfusion:</u><br>(1.RM.13, 1.KT.51, 5.PC.91.LA, 5.PC.91.HV) + CIHI BTREDBC = 1<br><br><u>Surgical or manual correction of inverted uterus for vaginal births only:</u><br>5.PC.91.HQ or 5.PC.91.HP, restricted to vaginal births (i.e., absence of caesarean 5.MD.60)<br><br><u>Reclosure of caesarean wound with RBC transfusion:</u><br>(5.PC.80.JM, 5.PC.80.JH) + CIHI BTREDBC = 1	

© 2020 Davidson AJF et al. *JAMA Network Open.*

Assessment	Timing	Disease, procedure or condition	ICD-10-CA or CCI codes, or other variables, in CIHI-DAD or MOMBABY	OHIP ICD-9 diagnostic codes or fee codes {or other source if in parentheses}
			Curettage with RBC transfusion: (5.PC.91.GA, 5.PC.91.GC, 5.PC.91.GD) + CIHI BTREDBC = 1<br><br>Maternal ICU admission: SCU in ('10', '20', '25', '30', '35', '40','45','60','80')	
Covariates	Up to 90 days before the estimated date of conception	Preconception hemoglobin A1c	--	LOINC code "17855-8", "17856-6", "41995-2", "4548-4", "71875-9", "59261-8" in {OLIS}
	Test taken closest to preconception A1c count used	Total hemoglobin concentration	--	LOINC code "718-7" in {OLIS}
	At the time of the early to mid-pregnancy A1c test	Gestational week	Gestational week at birth minus ([date of birth minus OBSERVATIONDATE]*7)	OBSERVATIONDATE in {OLIS}
	At the estimated date of conception	Maternal age	--	{RPDB}
	Same as above	Body mass index[a]	--	{BORN-Niday (April 2006-March 2012) & BORN-BIS (April 2012-March 2014)}
Stratified groups	Up to 90 days before the estimated date of conception	Preconception hemoglobin A1c	--	LOINC code "17855-8", "17856-6", "41995-2", "4548-4", "71875-9", "59261-8" in {OLIS}
Other baseline variables	Within one year preceding the estimated date of conception	Tobacco or drug dependence	291, 292, 2940, 303, 304, 305, 648.3, 649.0, 6555, 980 [F10-F19, F55, G312, O354, O355, T51, T652, Z720, Z721, Z722]	291, 292, 303, 304, 305
	Same as above	Chronic hypertension	401, 405, 642.0-642.2, 642.7 [I10, I15, O10, O11]	401
	Same as above	Serum creatinine concentration	--	LOINC code "14682-9" in {OLIS}

© 2020 Davidson AJF et al. *JAMA Network Open.*

Assessment	Timing	Disease, procedure or condition	ICD-10-CA or CCI codes, or other variables, in CIHI-DAD or MOMBABY	OHIP ICD-9 diagnostic codes or fee codes {or other source if in parentheses}
	Same as above	Renal disease	E1020, E1021, E1023, E1120, E1121, E1123, I12, I13, I150, I1701, M1039, M310, N01, N03-N08, N11, N12, N137-N139, N14-N19, N250, N258, N259, N26, O084, O904, R80, R944, T795,	403 581 585
	At the estimated date of conception	World region of origin	--	Country of birth {IRCC Permanent Resident Database Database}
	Same as above	Rural, urban residence	--	{Statistics Canada Census data}
	Same as above	Residential income quintile	--	{Statistics Canada Census data}
	At the index delivery	Multifetal pregnancy	Z37.2-Z37.7, Z37.90, O30, O31 (DAD delivery record) or Z38.3-Z38.6, Q89.4 (DAD newborn record)	--
	Same as above	Nulliparity or parity	Previous term deliveries + Previous pre-term deliveries (DAD delivery record)	--
	Same as above	Stillbirth	O364, Z371, Z373, Z374, Z376, Z377, P95	--

[a]Available for births between April 2006 and March 2014.

BIS: BORN Information System; BORN: Better Outcomes Registry & Network; CCI: Canadian Classification of Interventions; DAD: Discharge Abstract Database; ICD-9: International Classification of Diseases, 9th Revision; ICD-10-CA: International Classification of Diseases, 10th Revision, Canada; IRCC: Immigration, Refugees and Citizenship Canada; ODD: Ontario Diabetes Dataset; OHIP: Ontario Health Insurance Plan; OLIS: Ontario Laboratories Information System

© 2020 Davidson AJF et al. *JAMA Network Open.*

eTable 2. Classification of the 497 Infant Congenital Anomalies Diagnosed In The First Year Of Life. Congenital anomalies were grouped according to the International Classification of Diseases, 10th Revision, Canada. An individual pregnancy may have resulted in more than one type of congenital anomaly.

Type of congenital anomaly	Number (%) of affected pregnancies
Cardiac	237 (47.7)
Musculoskeletal system	89 (17.9)
Digestive system	82 (16.5)
Urinary system	49 (9.9)
Other congenital anomalies	39 (7.8)
Genital organs	35 (7.0)
Nervous system	31 (6.2)
Eye, ear, face and neck	29 (5.8)
Respiratory system	17 (3.4)
Cleft lip and cleft palate	7 (1.4)

© 2020 Davidson AJF et al. *JAMA Network Open.*

eTable 3. Risk of Adverse Perinatal and Maternal Outcomes In Relation To A 0.5% Absolute Higher Preconception Maternal Hemoglobin A1c Concentration, Restricted To 587 Women With Pre-Pregnancy Diabetes Mellitus And A Recorded Pre-Pregnancy Body Mass Index (BMI) (*Additional Analysis 1*). Relative risks are adjusted for pre-pregnancy BMI, maternal age at conception and hemoglobin concentration closest to the time of preconception A1c measurement.

Outcome	No. (overall %) with outcome	Adjusted relative risk (95% CI)
Perinatal		
Any congenital anomaly[a]	80 (13.7)	1.02 (0.97 to 1.08)
Cardiac congenital anomaly[a]	39 (6.7)	0.99 (0.92 to 1.08)
Preterm birth	141 (24.0)	1.07 (1.03 to 1.11)
Extreme preterm birth	13 (2.2)	1.01 (0.89 to 1.15)
Stillbirth or neonatal death less than 28 days postpartum	11 (1.9)	1.18 (1.08 to 1.30)
Maternal		
Severe maternal morbidity or death arising from 21 weeks' gestation up to 42 days postpartum	34 (5.8)	1.11 (1.04 to 1.20)
Severe maternal morbidity or death arising from the index birth up to 42 days postpartum	16 (2.7)	1.05 (0.89 to 1.23)

[a] Excluding any chromosomal anomaly.

© 2020 Davidson AJF et al. *JAMA Network Open.*

eTable 4. Risk of Adverse Perinatal And Maternal Outcomes Per 0.5% Absolute Net Decline In Maternal Hemoglobin A1c Between The Preconception And Early To Mid-Pregnancy Periods, Restricted To 587 Women With Pre-Pregnancy Diabetes Mellitus And A Recorded Pre-Pregnancy Body Mass Index (BMI) (*Additional Analysis 2*). Relative risks are adjusted for preconception A1c, maternal age at conception, hemoglobin concentration closest to the time of preconception A1c measurement, the gestational age of A1c measurement in the early to mid-pregnancy period, and pre-pregnancy BMI.

**Outcome**	**No. (overall %) with outcome**	**Adjusted relative risk (95% CI)**
Perinatal		
Any congenital anomaly[a]	80 (13.7)	1.01 (0.87 to 1.17)
Cardiac congenital anomaly[a]	39 (6.7)	1.06 (0.87 to 1.30)
Preterm birth	141 (24.0)	0.87 (0.80 to 0.94)
Extreme preterm birth	13 (2.2)	0.93 (0.72 to 1.20)
Stillbirth or neonatal death less than 28 days postpartum[b]	11 (1.9)	0.78 (0.62 to 0.99)
Maternal		
Severe maternal morbidity or death arising from 21 weeks' gestation up to 42 days postpartum	34 (5.8)	0.86 (0.71 to 1.03)
Severe maternal morbidity or death arising from the index birth up to 42 days postpartum	16 (2.7)	1.05 (0.73 to 1.50)

[a] Excluding any chromosomal anomaly.

© 2020 Davidson AJF et al. *JAMA Network Open.*

eTable 5. Risk of Adverse Perinatal And Maternal Outcomes Per 0.5% Absolute Net Decline In Maternal Hemoglobin A1c Between The Preconception Period And 3 To 12 Weeks' Gestation Among 1424 Births In Women With Pre-Pregnancy Diabetes Mellitus (*Additional Analysis 4*). Relative risks are adjusted for preconception A1c, maternal age at conception, hemoglobin concentration closest to the time of preconception A1c measurement, and the gestational age of A1c measurement in the period between 3 and 12 week's gestation.

Outcome	No. (overall %) with outcome	Adjusted relative risk (95% CI)
Perinatal		
Any congenital anomaly (N = 1422)[a]	201 (14.1)	0.90 (0.83 to 0.96)
Cardiac congenital anomaly (N = 1422)[a]	105 (7.4)	0.87 (0.80 to 0.94)
Preterm birth (PTB)	328 (23.0)	0.95 (0.91 to 1.01)
Extreme PTB < 32 weeks (N = 1424)	43 (3.0)	0.92 (0.81 to 1.04)
Stillbirth or neonatal death less than 28 days postpartum (N = 1422)	32 (2.3)	0.96 (0.81 to 1.12)
Maternal		
Severe maternal morbidity or death arising from 21 weeks' gestation up to 42 days postpartum (N = 1424)	66 (4.6)	0.90 (0.81 to 0.99)
Severe maternal morbidity or death arising from the index birth up to 42 days postpartum (N = 1424)	30 (2.1)	0.91 (0.68 to 1.11)

[a] Excluding any chromosomal anomaly.

© 2020 Davidson AJF et al. *JAMA Network Open.*
